# Circulating Ism1 Reduces the Risk of Type 2 Diabetes but not Diabetes-Associated NAFLD

**DOI:** 10.3389/fendo.2022.890332

**Published:** 2022-05-30

**Authors:** Jiajia Wang, Juan Du, Xiaoxu Ge, Wenfang Peng, Xirong Guo, Wenyi Li, Shan Huang

**Affiliations:** Department of Endocrinology, Tongren Hospital, Shanghai Jiao Tong University School of Medicine, Shanghai, China

**Keywords:** Ism1, type 2 diabetes, NAFLD, diabetes-associated NAFLD, adipokine

## Abstract

**Purpose:**

To examine the association of serum Ism1, a new adipokine that can regulate glucose uptake, with type 2 diabetes (T2D) in a Chinese population. Considering high prevalence of Nonalcoholic Fatty Liver Disease in patients with type 2 diabetes and the regulating role of Ism1 on glucose uptake of peripheral tissues, we further explored the association between Ism1 and diabetes-associated nonalcoholic fatty liver disease.

**Methods:**

A total of 120 newly diagnosed T2D patients and 60 control subjects with normal glucose were recruited in the case-control study. Serum Ism1 concentrations were determined by ELISA. Multivariate logistic regression analysis was used to evaluate the independent association of serum Ism1 concentration with the risk of T2D. The 120 newly diagnosed T2D patients were divided into uncomplicated T2D group and diabetes-associated NAFLD group according to the FLI score.

**Results:**

The Ism1 level of normoglycemic controls was higher than that of T2D patients (3.91 ± 0.24 ng/ml vs 3.01 ± 0.16 ng/ml, *P*=0.001). Based on quartile analysis of Ism1 level, the proportion of high circulating Ism1 levels in the control group increased while T2D group decreased, and the distribution difference was statistically significant (P=0.015). Logistic regression analysis indicated that the serum Ism1 level was an independent protective factor of type 2 diabetes (OR=0.69, 95%CI: 0.54-0.89). The decrease of Ism1 level did not increase the risk of non-alcoholic fatty liver disease in diabetic patients by Binary logistic regression analysis (OR=1.08, 95% CI: 0.69-1.69).

**Conclusions:**

The increase of serum Ism1 was associated with a decreased risk of diabetes, and it did not reduce the risk of non-alcoholic fatty liver disease in diabetic patients.

## Introduction

Diabetes is one of common and frequent metabolic diseases and has become the world’s third-largest chronic disease (1). The number of adults with diabetes between 20 and 79 years old worldwide has reached 463 million, and an estimated 693 million people globally will be suffering from diabetes by 2045 according to the International Diabetes Federation Survey (2). In China about 129.8 million diabetics account for nearly 12.8% of the total population, making the country become the most individuals and fastest-growing one in the world for diabetes (3). Such a high incidence of diabetes imposes a great burden not only to the individual but also to their family, society, and the healthcare system (4).

Type 2 diabetes is a metabolic disorder accompanied by increased insulin resistance and high blood glucose levels partly due to impaired glucose uptake and utilization by skeletal muscle, liver and adipose tissue (5, 6). Obesity is well known to be a significant risk factor for type 2 diabetes, and the increase in abdominal adipose tissue mass contributes to abnormal secretion of adipokines, which exerts important effects on glucose and lipid metabolism, insulin resistance and diabetes (7). To date, a variety of adipokines related to type 2 diabetes have been discovered and identified, including Adiponectin, Leptin, Visfatin, IL-6 and FGF21, etc (8, 9). However, the current research does not completely understand the mechanism of adipokines and new bioactive molecules remain to be discovered to predict and elucidate the pathogenesis of T2D and lead to the discovery of relevant biomarkers. Among these “adipokines” Isthmin-1 (Ism1) has received increasing attention recently.

Ism1 is a novel secreted protein unique to chordates, originally identified in the isthmus of Xenopus laevis during the early brain development in 2002 (10), and it is highly expressed in thyroid, placenta and adipose tissue (11). Previous studies have shed light on the multiple biological functions of Ism1 in embryonic development, anti-angiogenesis activity, inhibiting tumor growth and promoting apoptosis (12, 13). Recent advances have illuminated that Ism1 can regulate the glucose uptake of adipose tissue and reduce hepatic lipid synthesis in mice (14). However, to our knowledge, no one has focused on the correlation between Ism1 and T2D. Therefore, we intended to examine the difference in serum levels of Ism1 from newly-onset type 2 diabetes and normoglycemic controls, and analyzed the relationship between Ism1 level and the risk of diabetes by logistic regression.

## Method

### Study Design and Data Collection

This case-control study was approved by the Institutional Review Board of Shanghai Jiao Tong University School of Medicine Affiliated Tongren Hospital and conducted at the Department of Endocrinology from March to October 2021. Written informed consent was obtained from all participants.

The study included participants who were newly diagnosed with type 2 diabetes, based on the international classification of diabetes (ICD 10) criteria. The 120 newly diagnosed T2D patients were over 40 years old (mean age, 46.75 ± 1.20 years; BMI, 26.59 ± 0.54 kg/m^2^; males: females,72:48). At the same time, from the physical examination population in the same area, 60 healthy controls with normal blood glucose tolerance, HbA1c<5.6% and no history of endocrine and metabolism diseases were selected based on the frequency matching of age and BMI (mean age, 45.90 ± 0.98 years; BMI, 27.11 ± 0.45 kg/m^2^; males: females,37:23). In addition, all research subjects are of Han nationality.

### Detection of Serum Ism1and Insulin

Serum samples were collected from each participant and stored at -80°C. The serum levels of Ism1 were detected by Human Ism1 ELISA kits (JL14441, j&l biological, China). Insulin ELISA kits (10-1113-01, Mercodia, Sweden) were used to analyze the insulin level.

### Diagnosis of NAFLD

FLI, a non-invasive approach for diagnosis of nonalcoholic fatty liver disease was used in our research. The score of FLI ≥60 was considered to have NAFLD. The exclusion criteria are as follows: 1) heavy drinkers (men drink more than 140g per week and women drink more than 70g per week); 2) patients with a history of chronic liver disease.

### Statistical Analysis

SPSS 26 statistical software was used for clinical data analysis and statistics. All categorical variables are expressed as percentages. The continuous variables are expressed as the mean ± standard deviation if the data followed a normal distribution, while skewed distributed data are displayed as the median (interquartile range). Between-group comparisons for non-diabetic and diabetic groups were done with t-test, Mann-Whitney U test and chi-square test. *P* values<0.05 were considered as significant. Logistic regression analysis was used to analyze the correlation between Ism1 and T2D.

## Result

### Clinical and Laboratory Characteristics of the Controls and Type 2 Diabetes Population

A total of 120 participants who were diagnosed with new-onset diabetes were included in the case subjects and matched with 60 healthy controls subjects in Chinese population. Baseline characteristics of the T2D and control participants were summarized in **Table 1**. They were similar with respect to basic characteristics such as age, sex, height, weight and body mass index (BMI). Several biochemical indicators including uric acid (UA), lipids (TC, TG) and low-density lipoprotein (LDL-c) were not significantly different between the two groups. However, other biochemical parameters behaved differently. Except for high-density lipoprotein (HDL-c), creatinine that were higher in controls, T2D cases had higher levels of liver enzyme (ALT), fasting glucose (FPG) and fasting insulin (FINS). As expected, higher HOMA-IR (4.19 Vs 1.20) but lower HOMA-β (65.93 Vs 89.40) were observed in T2D than in control subjects (P<0.05), which means insulin resistance and β-cell function impairment occurred in diabetes cases.

**Table 1:** Clinical characteristics of the non-diabetic control group and T2D group.

Characteristic	Non-T2D group (n = 60)	T2D group (n = 120)	*P* value
Age (years)	46.75 ± 1.20	45.90 ± 0.98	0.946
Sex (M/F)	37/23	72/48	0.829
Height (m)	1.68 ± 0.01	1.69 ± 0.01	0.779
Weight (kg)	75.16 ± 1.73	77.62 ± 1.55	0.501
BMI (kg/m^2^)	26.59 ± 0.54	27.11 ± 0.45	0.492
FPG (mmol/L)	4.87 ± 0.05	8.63 ± 0.34	<0.001^**^
FINS (mU/L)	7.44 ± 0.71	14.87 ± 1.01	<0.001^**^
Cr (μmol/L)	65.84 (54.00-76.25)	56.50 (46.00-65.00)	<0.001^**^
UA (μmol/L)	360.50 (309.75-411.50)	302.08 (356.50-410.68)	0.728
TC (mmol/L)	4.99 (4.29-5.48)	4.91 (4.25-5.55)	0.592
TG (mmol/L)	1.52 (0.91-2.26)	1.69 (1.11-2.54)	0.143
LDL-c (mmol/L)	3.21 (2.71-3.68)	3.35 (2.69-3.86)	0.263
HDL-c (mmol/L)	1.17 (0.88-1.41)	1.00 (0.84-1.13)	<0.001^**^
ALT (U/L)	20 (14.25-33.00)	28.5 (19.00-53.25)	0.001^**^
HOMA-β	89.40 (58.16-146.03)	65.93 (33.04-136.71)	0.033^*^
HOMA-IR	1.20 (0.79-2.28)	4.19 (2.69-6.83)	0.001^**^
Ism1 (ng/mL)	3.72 (2.58-4.98)	3.01 (1.77-3.57)	0.001^**^

Data are expressed as mean ± SEM, median (interquartile range), or percentage values (%).

T2D, type 2 diabetes; BMI, body mass index; FPG, fasting plasma glucose; FINS, fasting plasma insulin; Cr, creatinine; UA, uric acid; TC, total cholesterol; TG, triglycerides; HDL-c, high density lipoprotein; LDL-c, low-density lipoprotein; Ism1, Isthmin-1; ALT, Alanine aminotransferase; HOMA-β, Homeostasis model assessment-β; HOMA-IR, homeostasis model assessment of insulin resistance. P values based on the chi-square test for the gender, Student’s t-test and Mann-Whitney test for other continuous variable. **P value <0.01. *P value <0.05 was considered significant.

### Comparison of Ism1 Level Between Diabetes and Control Groups

Much to our surprise, we noticed that the mean of serum Ism1 concentration (CV=4.1%) decreased in T2D group (3.01 ± 0.16 ng/ml) as compared to control group (3.91 ± 0.24 ng/ml), and the difference was statistically significant (*P*<0.001). To assess whether the decreased expression of serum Ism1 influenced the risk of diabetes, high and low expression levels were categorized based on quartiles. As shown in **Table 2**, the proportion of low circulating Ism1 levels (<3.02 ng/ml) was higher in the diabetes group than the non-diabetic groups (*P*=0.015).

**Table 2:** Distribution of Ism1 in the case and control groups.

Ism1 level classification (quartile)	Non-T2D group (n = 60)	T2D group (n = 120)
<P25 (<2.01 ng/mL)	9 (15.00)	36 (30.00)
P25~P49 (2.01 ng/mL~)	11 (18.33)	34 (28.33)
P50~ P75 (3.02 ng/mL~)	19 (31.67)	26 (21.67)
>P75 (≥4.16 ng/mL)	21 (35.00)	24 (20.00)

χ^2=^ 10.40 P=0.015, Data are expressed as number of samples, n (%).

### Binary Logistic Regression Analysis of Circulating Concentrations of Ism1, ALT, HLD-c and T2D

**Figure 1:**
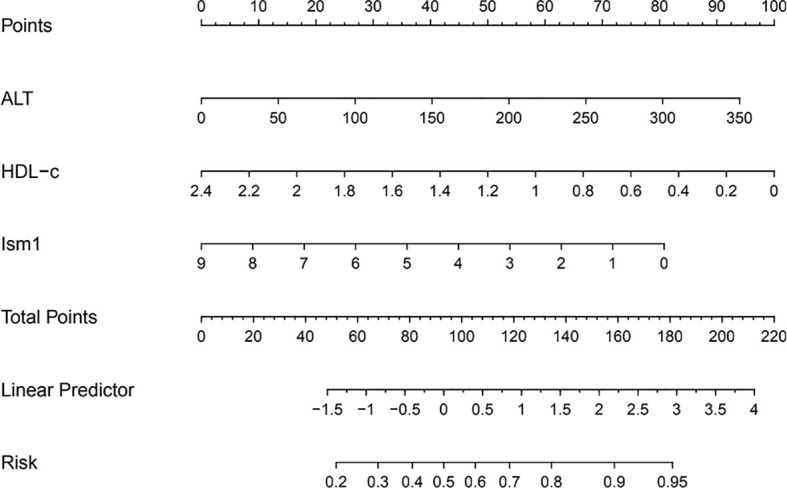
Ism1 and the risk of type 2 diabetes.

A multivariate analysis was performed to calculate OR value to explore the link between different variables and the occurrence of diabetes. Ism1, ALT a nd HLD-c were included in the regression when they were found to be significant (*P*<0.05). The levels of Ism1 was found to be an independent risk factor for T2D (OR=0.69, 95% CI=0.54-0.89, *P*=0.004), whereas ALT and HLD-c were no significant correlation with T2D (**Table 3**). Further, the association remained also when Ism1 was stratified by quartile, and the highest quartile level Ism1 was used as a reference. The results implied that T2D risk appeared to be significantly higher in subjects with the lowest quartile of Ism1 than in the subjects with the highest quartile of Ism1 (OR=4.69, 95% CI=1.22-17.93, *P*=0.024). However, compared with the highest quartile level Ism1, the second and third quartiles of Ism1 showed no significant correlation with T2D. To visualize the risk of ISM1 and type 2 diabetes, we created a nomogram (**Figure 1**).

**Table 3:** OR (95% CI) of T2D associated with ALT, HDL-c and the quartiles of Ism1.

Variable	β	Wald	*P* value	OR (95% CI)
ALT	0.009	0.13	0.72	1.00 (0.99-1.01)
HDL-c	-0.67	1.16	0.28	0.51 (0.15-1.73)
Continuous (Ism1)	-0.30	0.005	0.004^**^	0.69 (0.54-0.89)
Ism1 (<P25)	1.55	5.09	0.024^*^	4.69 (1.22-17.93)
Ism1 (P25~P49)	1.39	5.46	0.019^*^	4.86 (0.23-3.20)
Ism1 (P50~P75)	0.26	0.20	0.66	1.29 (0.42-4.02)
Ism1 (>P75)	/	8.87	0.031^*^	1.00

OR, odds ratio; CI, confidence interval; Values are given as OR (95% CI). **P value <0.01. *P value <0.05 was considered significant.

### Comparison of Baseline Clinical Characteristics of T2D Patients With or Without NAFLD

T2DM is closely linked to enhanced risk of developing nonalcoholic fatty liver disease, and it had been reported that more than 50% of T2D patients were accompanied by NAFLD (15, 16). As of now, relatively less is known about the prevalence of and risk factors for NAFLD with Type 2 diabetes in China; hence, further research is required in this area. As stated above, Low levels of Ism1 have been indicated as an independent risk factor for the development of T2D. The relationship between serum Ism1 levels and the development of NAFLD in diabetic patients was evaluated subsequently. FLI, a non-invasive approach for diagnosis of nonalcoholic fatty liver disease was used in our research. The score of FLI ≥60 was considered to have NAFLD. Among 120 diabetic patients, 48 patients were diagnosed with diabetes-associated NAFLD, so we matched the same number of uncomplicated diabetic cases and carried out our case-control study. There was no difference in the age and proportion of gender between the two groups of cases. Obese persons were more likely to suffer from NAFLD, especially those with abdominal obesity. As depicted in **Table 4**, this was indeed the case. Diabetes-associated NAFLD subjects were more likely to have abdominal obesity, with BMI of 30.97 kg/m2 and waist circumference of 103.18 cm. ALT, UA and FINS were significantly higher in diabetes-associated NAFLD than diabetes. Total cholesterol, LDL-c, and HDL-c levels did not show any significant change, whereas triglycerides levels were elevated. As we know, the most important link between T2D and NAFLD is insulin resistance (IR) (17, 18), HOMA-IR levels showed an elevation in subjects with diabetes-associated NAFLD. Of note, there was no significant change in circulating Ism1 levels in either group.

**Table 4:** Clinical characteristics of the T2D group and T2D&NAFLD group.

Characteristic	T2D group (n = 48)	T2DM&NAFLD group (n = 48)	*P* value
Age (years)	43.29 ± 1.32	42.54 ± 1.54	0.56
Sex (M/F)	29/19	31/17	0.67
BMI (kg/m^2^)	23.99 ± 0.49	30.97 ± 0.54	<0.001^**^
WC(cm)	87.21 ± 1.52	103.18 ± 1.24	<0.001^**^
FPG (mmol/L)	9.17 ± 0.52	8.60 ± 0.50	0.45
FINS (mU/L)	10.70 ± 0.83	18.80 ± 2.02	<0.001^**^
Cr (μmol/L)	54.30 (47.00-60.93)	59.00 (43.00-65.00)	0.39
UA (μmol/L)	350.50 (287.75-396.50)	370.50 (331.25-442.40)	0.036^*^
TC (mmol/L)	4.75 (4.11-5.56)	4.96 (4.56-5.57)	0.13
TG (mmol/L)	1.39 (0.88-2.16)	2.37 (1.67-3.35)	<0.001^**^
LDL-c (mmol/L)	3.34 (2.58-3.86)	3.31 (2.69-3.81)	0.95
HDL-c (mmol/L)	1.01 (0.89-1.21)	0.94 (0.83-1.03)	0.023^*^
ALT (U/L)	23.00 (15.25-45.70)	40.50 (25.00-65.25)	0.002^**^
HOMA-β	43.71 (23.27-82.24)	76.80 (40.51-171.41)	0.005^**^
HOMA-IR	3.90 (2.08-5.40)	5.55 (3.54-8.78)	0.001^**^
Ism1 (ng/mL)	2.50 (1.42-3.08)	2.87 (1.84-3.48)	0.13

T2D&IGR, T2D and NAFLD groups combined. Fatty liver index, FLI ≥60 was considered to have NAFLD, FLI <60, no NAFLD. WC: waist circumference. Data are expressed as mean ± SEM, median (interquartile range), or percentage values (%). **P value <0.01. *P value <0.05 was considered significant.

### Risk Factors for Diabetes-Associated Nonalcoholic Fatty Liver Disease.

The independent risk factors for concurrent diabetes-associated NAFLD were identified by a multivariate stepwise logistic regression analysis. Variables were included in the regression when they were found to be significant (*P*<0.05). Numerous studies have been confirmed that metabolic comorbidities such as obesity, type 2 diabetes, dyslipidemia and hypertension were strongly related to the prevalence and incidence of NAFLD (17, 19). In the present study, we found that BMI (OR=1.77, 95% CI=1.39-2.25, *P*<0.001) and TG (OR=1.72, 95% CI=1.02-2.88, *P*=0.04) were risk factors for NAFLD (**Table 5**). In addition, our results illustrated that low Ism1 levels did not increase the risk of NAFLD (OR=1.08, 95% CI=0.69-1.69, P=0.74) in diabetic patients.

**Table 5:** Binary Logistic regression analysis between level of Ism1 and T2D&NAFLD.

Variable	β	Wald	*P* value	OR (95% CI)
Ism1	0.08	0.11	0.74	1.08 (0.69-1.69)
BMI	0.57	21.45	<0.001^**^	1.77 (1.39-2.25)
ALT	-0.006	0.25	0.62	0.99 (0.97-1.02)
UA	0.003	0.42	0.52	1.00 (0.99-1.01)
TG	0.54	4.21	0.04^*^	1.72 (1.02-2.88)

Values are given as OR (95% CI). **P value <0.01. *P value <0.05 was considered significant.

## Discussion

Type 2 diabetes is a multifactorial metabolic disease that requires life-long therapy (20, 21). Although the early diagnosis of T2D is particularly important, there is still a lack of strong and effective biomarkers in the prediction of type 2 diabetes (22, 23). As such, we have explored the independent association of circulating Ism1 with the risk of T2D. To our knowledge, this is the first case-control study of Ism1 in population. Our results first demonstrated that a significant reduction of serum Ism1 level in T2D subjects when compared with control subjects. Logistic regression analysis indicated that this adipokine Ism1 is an independent protective factor for the development of T2D.

The biologically active adipokines secreted by adipose tissue have been frequently reported due to their main role in regulating metabolism, inflammation and glycolipid homeostasis to protect individuals from metabolic diseases onset (24, 25). So far, more than 600 adipokines have been proposed and the search for novel adipokines as biomarkers was still a hot topic (24). Among these adipokines that have been discovered, adiponectin and leptin were the most studied in the metabolism of glucose and lipids. Animal experimental studies have shown that adiponectin exerts its effects on insulin sensitivity mainly through activating AMPK cascade or PPAR-α, which leads to a reduction in the triglyceride content in liver and muscle, thus adiponectin has been recognized as one of the strongest biomarker of type 2 diabetes in numerous studies (26, 27). In the meta-analysis published at JAMA in 2009, an inverse association between adiponectin levels and T2D risk across diverse populations was confirmed by Li et al. (28). In line with Li, Wang et al. carried out a relatively large nested case-control study for the first time in a Chinese population, in which they prospectively evaluated the relationship between increased adiponectin and decreased T2D risk with 571 cases of diabetes and 571 age-sex-matched control cases (29). However, unlike adiponectin, the results from previous researches on the leptin-T2D association were not consistent. Several studies have shown a positive correlation of leptin concentrations with T2D risk (30–32). Conversely, an American prospective study reported that plasma leptin was unlikely to serve as a biomarker to predict future diabetes (33). Due to this, adiponectin appears to be more closely related to the risk of diabetes than leptin. For other adipokines including FGF21, IL-6 and resistin might help distinguish those at higher risk of developing T2D. Similar to other adipokines, Ism1 could regulate glucose tolerance, modulate insulin sensitivity by stimulating the PI3K-AKT signaling pathway independently of insulin and IGF receptors. Our data has confirmed that Ism1 was an independent protective factor for the development of T2D, so Ism1 and adipokines may be used to build models to predict the risk of diabetes in the population. Consequently, further prospective clinical trials were required to demonstrate the predictive value of Ism1 for identifying the risk of diabetes in the population.

NAFLD is more prevalent in individuals with conditions such as obesity, T2DM, and metabolic syndrome (34). Due to the dual roles of adiponectin in the pathogenesis of NAFLD and type 2 diabetes, adiponectin would represent a potential biomarker for the prediction of NAFLD and type 2 diabetes (35). Ebrahimi R et al. reported that lower level of adiponectin was closely involved in the development of T2D, NAFLD, and NAFLD&T2D independent of IR and obesity indices (36). In studies by others, FGF21, CTRP13 and A-FABP might be an associated factor for both NAFLD and dysglycemia (35, 37). Like adiponectin, Ism1 has a dual role in increasing adipocyte glucose uptake while suppressing hepatic lipid synthesis in mouse models (14), the potential of Ism1 to be an independent marker for NAFLD in patients with T2D was examined in this study. However, we found no apparent alterations in circulating Ism1 levels between diabetes and diabetes-associated NAFLD subjects, which did not support Ism1 as an independent risk factor for diabetes-associated NAFLD by logistic regression analysis.

There were a few shortcomings in this study, one of the limitations was that it was only a case-control study, and the sample size was not large enough, with only 180 cases. The study was not comprehensive enough and was not conducive to revealing the causative relationship between Ism1 and T2D. Therefore, further study should be evaluated by expanding the number of patients and implementing a prospective cohort. Another limitation was that this study was conducted in an age-dispersed population; therefore, research on Ism1 in elderly population with a higher T2D incidence still needs to be performed. In addition, we did not perform pathological diagnosis by using the traditional method for NAFLD, the inclusion criteria for NAFLD need to be further optimized.

In summary, our results demonstrated that the secretion of Ism1 was altered in subjects with T2D, but it was not found in patients with diabetes-associated NAFLD clearly suggesting that Ism1 was an independent protective factor for diabetes but not diabetes-associated NAFLD. Therefore, it can be a potentially useful new biomarker for the early diagnosis and management of diabetes.

## Data Availability Statement

The raw data supporting the conclusions of this article will be made available by the authors, without undue reservation.

## Ethics Statement

The studies involving human participants were reviewed and approved by The Ethics Committee of Tongren Hospital, Shanghai Jiao Tong University School of Medicine. The patients/participants provided their written informed consent to participate in this study. Written informed consent was obtained from the individual(s) for the publication of any potentially identifiable images or data included in this article.

## Author Contributions

XRG, WL and SH designed the research. JW performed the experiments and data analysis. JD and XXG collected the clinical data of participants and serum samples. WP assisted in research design and experiments. WL and JW prepared the paper. All authors contributed to the article and approved the submitted version.

## Funding

This study was supported by National Natural Sciences Foundation of China Grants (81770769), Natural Science Foundation Project of Shanghai Scientific and technological innovation plan (No.22ZR1457000, National Key Research and Development Program of China (2021YFC2701900,2021YFC2701903), Master and Doctor innovation talent base for endocrine and metabolic diseases (RCJD2021S03), Key laboratory for translational research and innovative therapeutics of gastrointestinal oncology(ZDSYS-2020-05).

## Conflict of Interest

The authors declare that the research was conducted in the absence of any commercial or financial relationships that could be construed as a potential conflict of interest.

## Publisher’s Note

All claims expressed in this article are solely those of the authors and do not necessarily represent those of their affiliated organizations, or those of the publisher, the editors and the reviewers. Any product that may be evaluated in this article, or claim that may be made by its manufacturer, is not guaranteed or endorsed by the publisher.

## References

[1] Ali MK Singh K Kondal D Devarajan R Patel SA Shivashankar R. Effectiveness of a Multicomponent Quality Improvement Strategy to Improve Achievement of Diabetes Care Goals: A Randomized, Controlled Trial. Ann Intern Med (2016) 165(6):399–408. doi: 10.7326/M15-2807 27398874 PMC6561084

[2] Cho NH Shaw JE Karuranga S Huang Y da RochaFernandes JD Ohlrogge AW. Idf Diabetes Atlas: Global Estimates of Diabetes Prevalence for 2017 and Projections for 2045. Diabetes Res Clin Pract (2018) 138:271–81. doi: 10.1016/j.diabres.2018.02.023 29496507

[3] Li Y Teng D Shi X Qin G Qin Y Quan H. Prevalence of Diabetes Recorded in Mainland China Using 2018 Diagnostic Criteria From the American Diabetes Association: National Cross Sectional Study. BMJ (2020) 369:m997. doi: 10.1136/bmj.m997 32345662 PMC7186854

[4] Wang T Lu J Shi L Chen G Xu M Xu Y. Association of Insulin Resistance and β-Cell Dysfunction With Incident Diabetes Among Adults in China: A Nationwide, Population-Based, Prospective Cohort Study. Lancet Diabetes Endocrinol (2020) 8(2):115–24. doi: 10.1016/S2213-8587(19)30425-5 31879247

[5] Type 2 Diabetes Mellitus. Nat Rev Dis Primers (2015) 1(1):15039. doi: 10.1038/nrdp.2015.39 27228210

[6] Ding L Houben T Oligschlaeger Y Bitorina AV Verwer BJ Tushuizen ME. Plasma Cathepsin D Activity Rather Than Levels Correlates With Metabolic Parameters of Type 2 Diabetes in Male Individuals. Front Endocrinol (Lausanne) (2020) 11:575070. doi: 10.3389/fendo.2020.575070 33101209 PMC7554511

[7] Luo J Hendryx M Laddu D Phillips LS Chlebowski R LeBlanc ES. Racial and Ethnic Differences in Anthropometric Measures as Risk Factors for Diabetes. Diabetes Care (2019) 42(1):126–33. doi: 10.2337/dc18-1413 PMC6463546 30352893

[8] Huang T Glass K Zeleznik OA Kang JH Ivey KL Sonawane AR. A Network Analysis of Biomarkers for Type 2 Diabetes. Diabetes (2019) 68(2):281–90. doi: 10.2337/db18-0892 PMC6341308 30409783

[9] Liao PJ Ting MK Wu IW Chen SW Yang NI Hsu KH. Higher Leptin-to-Adiponectin Ratio Strengthens the Association Between Body Measurements and Occurrence of Type 2 Diabetes Mellitus. Front Public Health (2021) 9:678681. doi: 10.3389/fpubh.2021.678681 34368053 PMC8342761

[10] Pera E Kim J Martinez S Brechner M Li S Wessely O. Isthmin Is a Novel Secreted Protein Expressed as Part of the Fgf-8 Synexpression Group in the Xenopus Midbrain-Hindbrain Organizer. Mech Dev (2002) 116:169–72. doi: 10.1016/S0925-4773(02)00123-5 12128218

[11] Berrun A Harris E Stachura DL. Isthmin 1 (ism1) is Required for Normal Hematopoiesis in Developing Zebrafish. PloS One (2018) 13(5):e0196872. doi: 10.1371/journal.pone.0196872 29758043 PMC5951578

[12] Osorio L Wu X Zhou Z. Distinct Spatiotemporal Expression of ISM1 During Mouse and Chick Development. Cell Cycle (2014) 13(10):1571–82. doi: 10.4161/cc.28494 PMC4050162 24675886

[13] Valle-Rios R Maravillas-Montero JL Burkhardt AM Martinez C Buhren BA Homey B. Isthmin 1 is a Secreted Protein Expressed in Skin, Mucosal Tissues, and NK, NKT, and th17 Cells. J Interferon Cytokine Res (2014) 34(10):795–801. doi: 10.1089/jir.2013.0137 24956034 PMC4186767

[14] Jiang Z Zhao M Voilquin L Jung Y Aikio MA Sahai T. Isthmin-1 is an Adipokine That Promotes Glucose Uptake and Improves Glucose Tolerance and Hepatic Steatosis. Cell Metab (2021) 33(9):1836–1852.e11. doi: 10.1016/j.cmet.2021.07.010 34348115 PMC8429235

[15] Alexopoulos AS Duffy R Kobe EA German J Moylan CA Soliman D. Underrecognition of Nonalcoholic Fatty Liver Disease in Poorly Controlled Diabetes: A Call to Action in Diabetes Care. J Endocr Soc (2021) 5(12):bvab155. doi: 10.1210/jendso/bvab155 34755002 PMC8570418

[16] Qi Y Fan L Ran D Xu J Wang Y Wu J. Main Risk Factors of Type 2 Diabetes Mellitus With Nonalcoholic Fatty Liver Disease and Hepatocellular Carcinoma. J Oncol (2021) 2021:7764817. doi: 10.1155/2021/7764817 34691178 PMC8528616

[17] Cariou B Byrne CD Loomba R Sanyal AJ. Nonalcoholic Fatty Liver Disease as a Metabolic Disease in Humans: A Literature Review. Diabetes Obes Metab (2021) 23(5):1069–83. doi: 10.1111/dom.14322 PMC8248154 33464677

[18] Hui ST Parks BW Org E Norheim F Che N Pan C. The Genetic Architecture of NAFLD Among Inbred Strains of Mice. Elife (2015) 4:e05607. doi: 10.7554/eLife.05607 26067236 PMC4493743

[19] Marchisello S Di Pino A Scicali R Urbano F Piro S Purrello F. Pathophysiological, Molecular and Therapeutic Issues of Nonalcoholic Fatty Liver Disease: An Overview. Int J Mol Sci (2019) 20(8). doi: 10.3390/ijms20081948 PMC6514656 31010049

[20] Chen A Huang Z Wan X Deng W Wu J Li L. Attitudes Toward Diabetes Affect Maintenance of Drug-Free Remission in Patients With Newly Diagnosed Type 2 Diabetes After Short-Term Continuous Subcutaneous Insulin Infusion Treatment. Diabetes Care (2012) 35(3):474–81. doi: 10.2337/dc11-1638 PMC3322723 22228747

[21] Zhou T Kim TW Chong CN Tan L Amin S Sadat Badieyan Z. A hPSC-based Platform to Discover Gene-Environment Interactions That Impact Human Beta-Cell and Dopamine Neuron Survival. Nat Commun (2018) 9(1):4815. doi: 10.1038/s41467-018-07201-1 30446643 PMC6240096

[22] Adua E Roberts P Wang W. Incorporation of Suboptimal Health Status as a Potential Risk Assessment for Type II Diabetes Mellitus: A Case-Control Study in a Ghanaian Population. EPMA J (2017) 8(4):345–55. doi: 10.1007/s13167-017-0119-1 PMC5700018 29209438

[23] Hulsegge G Spijkerman A van derSchouw Y Bakker S Gansevoort R Smit H. Trajectories of Metabolic Risk Factors and Biochemical Markers Prior to the Onset of Type 2 Diabetes: The Population-Based Longitudinal Doetinchem Study. Nutr Diabetes (2017) 7(5):e270. doi: 10.1038/nutd.2017.23 28481339 PMC5518805

[24] Bluher M Mantzoros CS. From Leptin to Other Adipokines in Health and Disease: Facts and Expectations at the Beginning of the 21st Century. Metabolism (2015) 64(1):131–45. doi: 10.1016/j.metabol.2014.10.016 25497344

[25] Frances L Tavernier G Viguerie N. Adipose-Derived Lipid-Binding Proteins: The Good, the Bad and the Metabolic Diseases. Int J Mol Sci (2021) 22(19):10460. doi: 10.3390/ijms221910460 34638803 PMC8508731

[26] Khoramipour K Chamari K Hekmatikar AA Ziyaiyan A Taherkhani S Elguindy NM. Adiponectin: Structure, Physiological Functions, Role in Diseases, and Effects of Nutrition. Nutrients (2021) 13(4):1180. doi: 10.3390/nu13041180 33918360 PMC8066826

[27] Uslu S Kebapci N Kara M Bal C. Relationship Between Adipocytokines and Cardiovascular Risk Factors in Patients With Type 2 Diabetes Mellitus. Exp Ther Med (2012) 4(1):113–20. doi: 10.3892/etm.2012.557 PMC3460256 23060933

[28] Li S Shin H Ding E van Dam R. Adiponectin Levels and Risk of Type 2 Diabetes: A Systematic Review and Meta-Analysis. JAMA (2009) 302(2):179–88. doi: 10.1001/jama.2009.976 19584347

[29] Wang Y Meng RW Kunutsor SK Chowdhury R Yuan JM Koh WP. Plasma Adiponectin Levels and Type 2 Diabetes Risk: A Nested Case-Control Study in a Chinese Population and an Updated Meta-Analysis. Sci Rep (2018) 8(1):406. doi: 10.1038/s41598-017-18709-9 29321603 PMC5762808

[30] Welsh P Murray HM Buckley BM de Craen AJ Ford I Jukema JW. Leptin Predicts Diabetes But Not Cardiovascular Disease: Results From a Large Prospective Study in an Elderly Population. Diabetes Care (2009) 32(2):308–10. doi: 10.2337/dc08-1458 PMC2628699 19001191

[31] Sahin-Efe A Upadhyay J Ko BJ Dincer F Park KH Migdal A. Irisin and Leptin Concentrations in Relation to Obesity, and Developing Type 2 Diabetes: A Cross Sectional and a Prospective Case-Control Study Nested in the Normative Aging Study. Metabolism (2018) 79:24–32. doi: 10.1016/j.metabol.2017.10.011 29108900

[32] Soderberg S Zimmet P Tuomilehto J Chitson P Gareeboo H Alberti KG. Leptin Predicts the Development of Diabetes in Mauritian Men, But Not Women: A Population-Based Study. Int J Obes (Lond) (2007) 31(7):1126–33. doi: 10.1038/sj.ijo.0803561 17325688

[33] Sun Q van Dam RM Meigs JB Franco OH Mantzoros CS Hu FB. Leptin and Soluble Leptin Receptor Levels in Plasma and Risk of Type 2 Diabetes in U.S. Women: A Prospective Study. Diabetes (2010) 59(3):611–8. doi: 10.2337/db09-1343 PMC2828671 19959759

[34] El-Ashmawy HM Ahmed AM. Serum Fetuin-B Level is an Independent Marker for Nonalcoholic Fatty Liver Disease in Patients With Type 2 Diabetes. Eur J Gastroenterol Hepatol (2019) 31(7):859–64. doi: 10.1097/MEG.0000000000001354 30601337

[35] Hui E Xu A Bo Yang H Lam KS. Obesity as the Common Soil of non-Alcoholic Fatty Liver Disease and Diabetes: Role of Adipokines. J Diabetes Investig (2013) 4(5):413–25. doi: 10.1111/jdi.12093 PMC4025109 24843689

[36] Ebrahimi R Shanaki M Mohassel Azadi S Bahiraee A Radmard AR Poustchi H. Low Level of Adiponectin Predicts the Development of Nonalcoholic Fatty Liver Disease: Is it Irrespective to Visceral Adiposity Index, Visceral Adipose Tissue Thickness and Other Obesity Indices? Arch Physiol Biochem (2019) 1–8. doi: 10.1080/13813455.2019.1661496 31482741

[37] Shanaki M Fadaei R Moradi N Emamgholipour S Poustchi H. The Circulating CTRP13 in Type 2 Diabetes and Non-Alcoholic Fatty Liver Patients. PloS One (2016) 11(12):e0168082. doi: 10.1371/journal.pone.0168082 27936230 PMC5148106

